# Evaluation of on‐ and off‐target effects of self‐assembled epidermal growth factor receptor small interfering RNA delivery system

**DOI:** 10.1002/ctm2.1579

**Published:** 2024-02-05

**Authors:** Hongyuan Guo, Yuanyuan Su, Ruoyan Zhang, Xiao Hu, Hao Zhu, Xin Yan, Chen‐Yu Zhang, Xu Guo, Zhen Zhou, Xi Chen

**Affiliations:** ^1^ Nanjing Drum Tower Hospital Center of Molecular Diagnostic and Therapy, State Key Laboratory of Pharmaceutical Biotechnology, Jiangsu Engineering Research Center for MicroRNA Biology and Biotechnology, NJU Advanced Institute of Life Sciences (NAILS), School of Life Sciences Nanjing University Nanjing China; ^2^ Chemistry and Biomedicine Innovation Center (ChemBIC) Nanjing University Nanjing China; ^3^ Research Unit of Extracellular RNA Chinese Academy of Medical Sciences Nanjing China; ^4^ Institute of Artificial Intelligence Biomedicine Nanjing University Nanjing China; ^5^ Institute of Basic Medical Sciences Chinese Academy of Medical Sciences and Peking Union Medical College Beijing China; ^6^ Department of Respiratory and Critical Care Medicine Nanjing Drum Tower Hospital, The Affiliated Hospital of Nanjing University Medical School Nanjing China; ^7^ Department of Vascular Surgery Nanjing Drum Tower Hospital, Affiliated Hospital of Medical School, Nanjing University Nanjing China

Dear Editor,

RNAi therapy holds inherent potential to address limitations associated with targeted anticancer drugs. However, the clinical advancement of small interfering RNA (siRNA)‐based therapeutics faces a significant challenge in the realm of in vivo delivery, particularly for extrahepatic targets. To overcome this obstacle, we have recently developed an innovative siRNA delivery strategy. This approach involves reprogramming the host liver using genetic circuits. The reprogramming triggers the synthesis of siRNAs, which subsequently self‐assemble into secretory small extracellular vesicles (sEVs) delivered to specific tissues through the circulating system. While the efficiency and efficacy of this delivery system have been demonstrated across various diseases,^1^, ^2^, ^3^, ^4^, ^5^ a critical aspect that remains insufficiently characterized is the on‐target and off‐target toxicity effects. Taking the epidermal growth factor receptor (EGFR) gene as a representative, we assessed the on‐target effects in tumour and normal cells after self‐assembled siRNA treatment both in vitro and in vivo. Further, we comprehensively evaluated the off‐target effects in tumours and main tissues with the prospect of addressing the potential concerns of the systemic siRNA delivery approach.

Following the described strategy,^1^ we constructed the cytomegalovirus (CMV)‐siR^E^ circuit, featuring an EGFR siRNA‐expressing module downstream of a CMV promoter. To demonstrate the inhibitory potential of EGFR siRNA‐encapsulating sEVs, HEK293T cells were transfected with CMV‐siR^E^ or CMV‐scrR, a control expressing scramble RNA. sEVs obtained from the culture medium of HEK293T cells using differential centrifugation were analyzed using dynamic light scattering, revealing a peak at 132 nanometers (Figure S1). The separation of sEVs into distinct layers was achieved through gradient ultracentrifugation (Figure S2A). Upon transmission electron microscopy analysis, vesicles exhibiting the characteristic size and morphology of sEVs were only observed in the middle layer, but not in the low and high layers (Figure S2B). Western blotting analysis indicated that the middle layer showed high enrichment for CD9, CD63 and TSG101, distinguishing it from the low and high layers where these sEV markers were absent (Figure S2C). In addition, a majority of the EGFR siRNAs were localized within the middle layer (Figure S2D). Furthermore, the sEVs were subjected to anti‐CD9 or anti‐CD63 immunoprecipitation, revealing a notably higher recovery of EGFR siRNA from the CD9‐ or CD63‐positive sEVs (Figure S2E,F). These results demonstrated the EGFR siRNA was mainly encapsulated in sEVs assembled from CMV‐siR^E^. Next, we incubated the cell‐assembled sEVs with human lung adenocarcinoma cells (H1975) or human bronchial epithelial cells (BEAS‐2B) (Figure 1A). A reduction in EGFR expression was observed in both cell lines along with the increase of sEVs (Figure 1B,C and Figure S3A,B) or synthetic EGFR siRNA doses (Figure 1D,E and Figure S3C,D). Notably, even a minimal amount of EGFR siRNAs (0.3 fM) encapsulated in sEVs downregulated EGFR protein in H1975 and BEAS‐2B cells by over 50%, mirroring the interference achieved by a large quantity of synthetic EGFR siRNAs (0.625 nM). Analysis of EGFR siRNA concentrations in sEV‐treated cells versus EGFR siRNA‐transfected cells revealed lower levels of siRNA in cells after co‐culture with sEVs (Figure 1F). Despite the EGFR siRNA delivered by sEVs being approximately 1/2,000,000 of synthetic transfection, it exhibited potent interference on EGFR protein. We hypothesized that the majority of transfected synthetic siRNAs, not actively loaded into functional Argonaute proteins, act at supraphysiological levels.^6^ In contrast, sEV‐enclosed siRNAs, processed endogenously and packaged into sEVs, were taken up in a physiological state, readily functioning in recipient cells. In alignment with the “oncogene addiction” theory,^7^ inhibition of EGFR protein expression by ∼50% induced significant apoptosis in H1975 cells but not in BEAS‐2B cells (Figure 2A–D). In contrast, synthetic EGFR siRNAs caused increased apoptosis in both cell lines (Figure 2E–H). These results indicate that the lower concentration of sEV‐enclosed EGFR siRNAs from the CMV‐siR^E^ circuit demonstrates superior biosafety to somatic cells compared to synthetic EGFR siRNAs.

**FIGURE 1:**
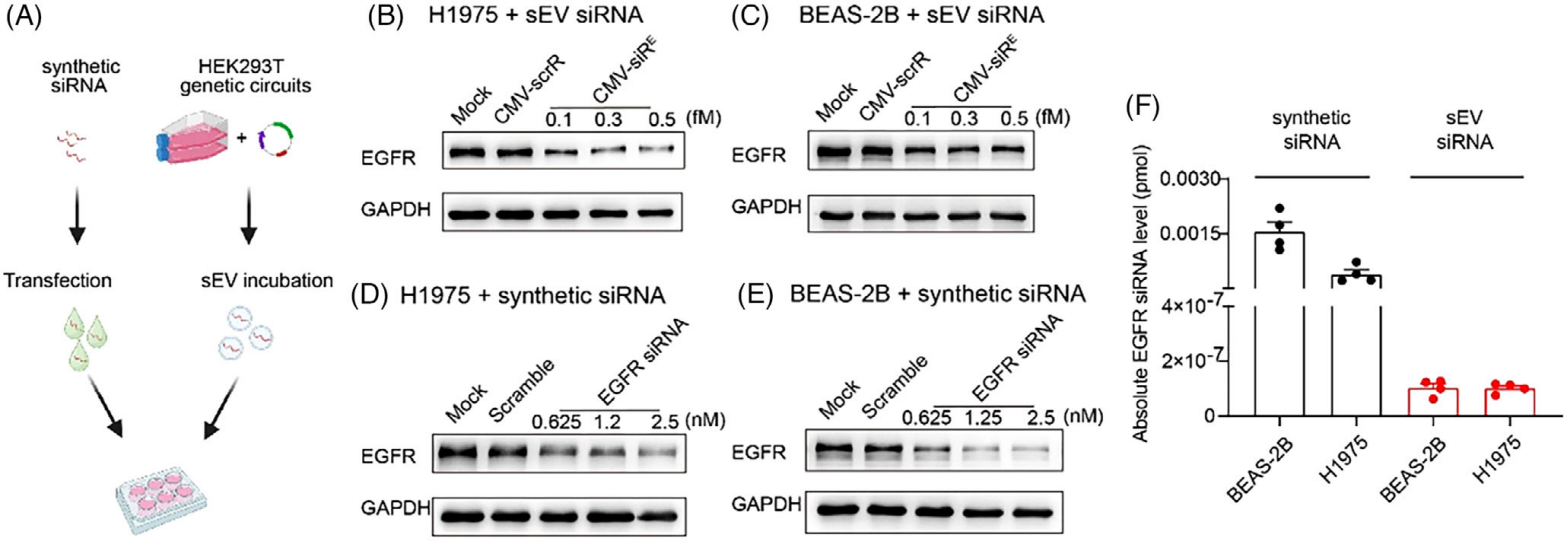
Evaluation of the silencing efficacy of epidermal growth factor receptor (EGFR) small interfering RNA (siRNA)**‐encapsulating** small extracellular vesicles (sEVs) **in vitro**. (A) Flow chart of the experimental design. HEK293T cells were transfected with cytomegalovirus (CMV)‐scrR or CMV‐siR^E^ circuits. At 36 h post‐transfection, sEVs were purified from the cell culture medium, and an increasing amount of sEVs were incubated with H1975 and BEAS‐2B cells. Meanwhile, an increasing amount of synthetic EGFR siRNAs were directly transfected into H1975 and BEAS‐2B cells with lipofectamine as a control. (B, C) Western blot analysis of EGFR protein levels in H1975 and BEAS‐2B cells incubated with sEVs derived from CMV‐scrR or CMV‐siR^E^ circuit‐transfected HEK293T cells. Different doses (EGFR siRNA concentration in culture medium was 0.1, 0.3 and 0.5 fM, respectively) of CMV‐siR^E^ derived sEVs were added to evaluate the inhibitory effects. CMV‐scrR‐derived sEVs (0.5 fM) were added as a negative control, and untreated H1975 and BEAS‐2B cells served as the mock controls. EGFR protein levels were determined 36 h post‐incubation. Representative Western blots (b, c) are shown (*n* = 5 per group). (D, E) Western blot analysis of EGFR protein levels in H1975 and BEAS‐2B cells transfected with synthetic EGFR siRNA. Different doses (EGFR siRNA concentration in culture medium was 0.625, 1.25 and 2.5 nM, respectively) of synthetic siRNA were added to evaluate the dose‐dependent effects. EGFR protein levels were determined 36 h post‐transfection. Synthetic scramble siRNA (2.5 nM) was transfected as a negative control, and untransfected H1975 and BEAS‐2B cells served as the mock controls. Representative Western blots (D, E) are shown (*n* = 5 per group). (F) Quantitative real‐time polymerase chain reaction (RT–PCR) analysis of the accumulation of EGFR siRNAs in recipient H1975 and BEAS‐2B cells after incubation with EGFR siRNA‐encapsulating sEVs or transfection with synthetic EGFR siRNAs (*n* = 4 per group).

**FIGURE 2:**
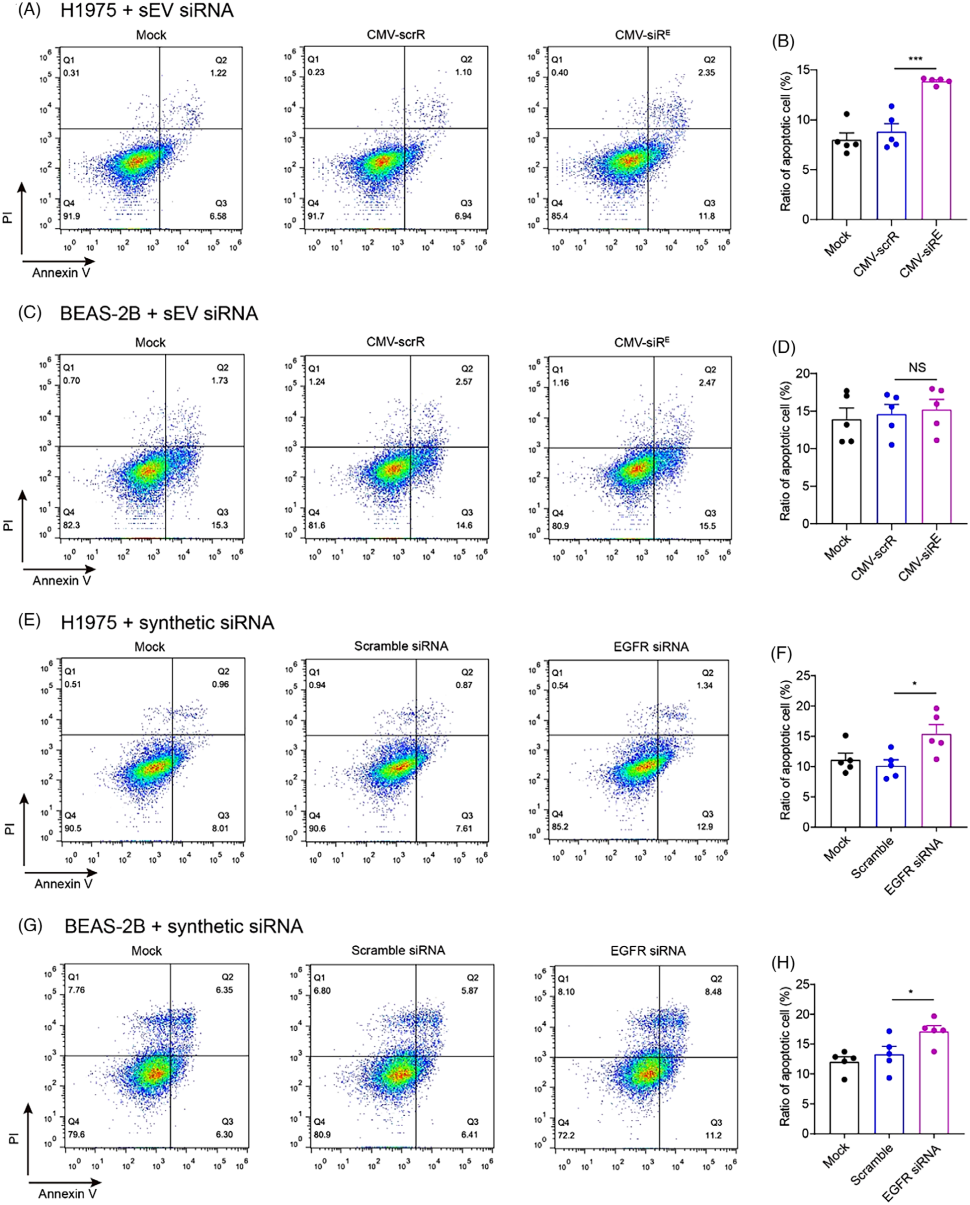
Comparison of the cellular apoptosis caused by sEV‐encapsulated and synthetic small interfering RNA (siRNA) **in vitro**. Apoptosis of the H1975 and BEAS‐2B cells incubated with epidermal growth factor receptor (EGFR) siRNA‐encapsulating small extracellular vesicles (sEVs) (A–D) or transfected with synthetic EGFR siRNAs (E–H) was analysed by flow cytometry after Annexin V/PI double staining. The percentage of apoptotic cells was calculated by dividing the sum of early apoptotic (PI^−^ Annexin^+^) and late apoptotic (PI^+^ Annexin^+^) cells by the total number of cells (*n* = 5 per group). Values are presented as the means ± SEM. Significance was determined using one‐way analysis of variance (ANOVA) followed by Dunnett's multiple comparison test in panels (B), (D), (F) and (H). *p < .05; **p < .01; ***p < .005; NS, not significant.

To assess the in vivo therapeutic efficacy and biosafety of sEV‐enclosed EGFR siRNAs, we utilized an orthotopic lung cancer mouse model established with the H1975 cell line. Following tumour confirmation via micro‐computed tomography (micro‐CT) scanning, mice were subjected to intravenous injection with phosphate‐buffered saline, CMV‐scrR or CMV‐siR^E^ circuit (10 mg/kg every 2 days) seven times (Figure 3A). In line with in vitro findings, sEV‐encapsulated EGFR siRNAs demonstrated potent anti‐tumour effects in serum at concentrations of approximately 1000–2000 fM, akin to endogenous circulating microRNAs (miRNAs) (Figure S4A). Micro‐CT imaging and 3‐D reconstructions revealed substantial attenuation of lung tumour growth in mice treated with the CMV‐siR^E^ circuit (Figure 3B,C). This result was corroborated by histopathological examination of lung tissues (Figure 3D). The examination of the H&E‐stained sections revealed a distinct alleviation in tumour size and a noticeable decrease in the nuclear‐to‐cytoplasmic ratio following the administration of the CMV‐siR^E^ circuit in comparison with control groups. Immunohistochemistry (IHC) staining confirmed lowered EGFR protein levels in CMV‐siR^E^‐treated tumours, accompanied by increased Caspase‐3 (Figure 3E–G). Both EGFR protein and mRNA were significantly downregulated in lung tumours treated with the CMV‐siR^E^ circuit (Figure 3H–J) with a substantial amount of EGFR siRNA detected in tumour tissue (Figure S4B). Evaluation of potential side effects on normal tissues revealed no signs of damage in the lung, liver, brain, heart and muscle upon repeated injection of the CMV‐siR^E^ circuit (Figure S5). IHC staining of Caspase‐3 in these tissues showed no apparent apoptotic signals (Figure S6). These results demonstrated significant anti‐EGFR effects and tumour apoptosis while exhibiting minimal toxicity in normal tissues.

**FIGURE 3:**
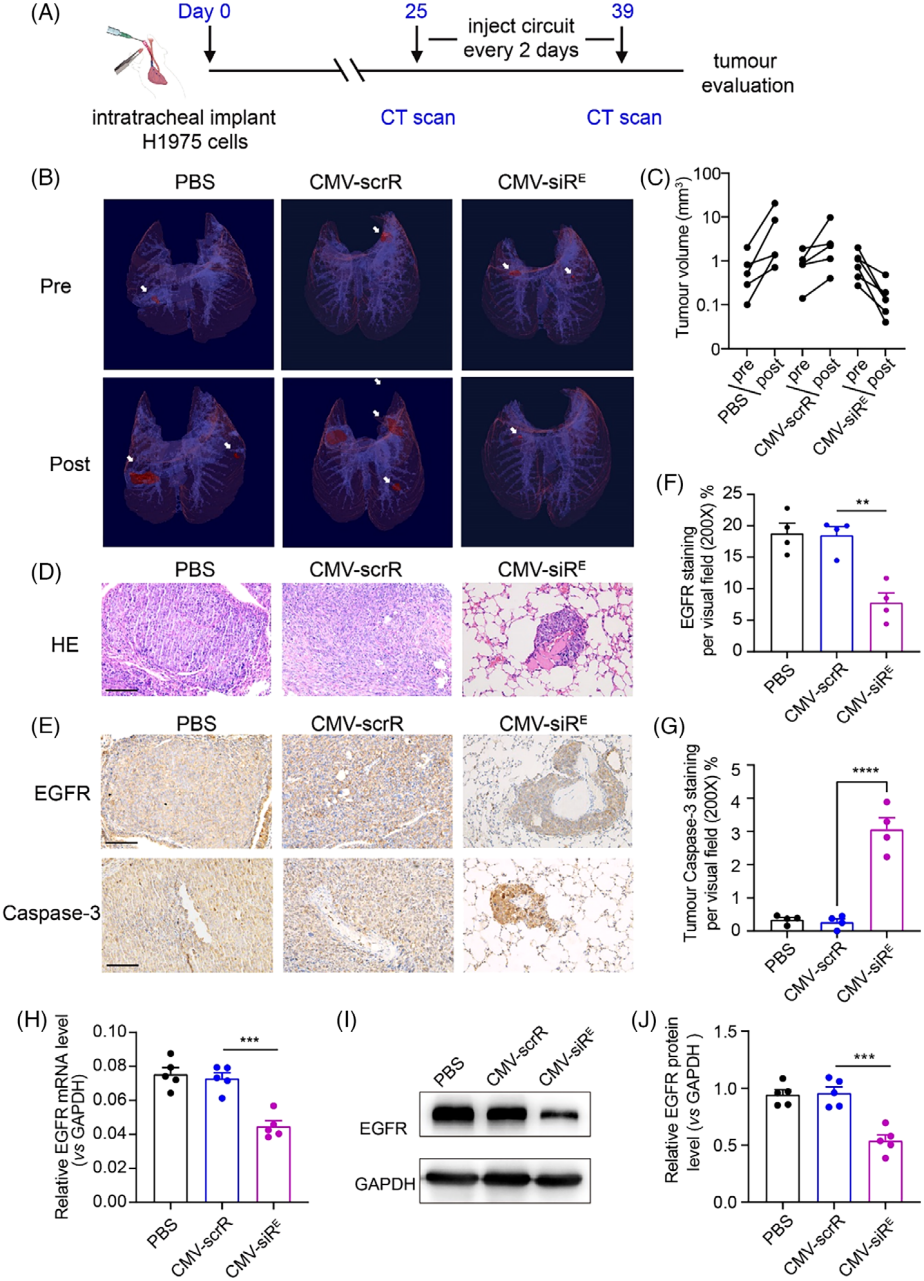
Evaluation of the therapeutic efficacy of self‐assembled epidermal growth factor receptor (EGFR) small interfering RNAs (siRNAs) **in an orthotopic lung cancer model**. (A) Flow chart of the experimental design. Nude mice were intratracheally implanted with H1975 cells and analysed by micro‐CT at 25 days post‐inoculation to ensure the formation of tumours in the lungs. Mice were then intravenously injected with phosphate‐buffered saline (PBS) or the cytomegalovirus (CMV)‐scrR or CMV‐siR^E^ circuit (10 mg/kg) every 2 days for a total of seven treatments. After treatment, tumour growth and EGFR expression levels were evaluated. (B) Representative 3‐D reconstructions of mouse lungs pre‐ and post‐treatment with genetic circuits. Tumours are shown in maroon to demonstrate their locations in the 3‐D reconstructions. (C) Semiautomated quantitative image analysis was performed using the 3‐D reconstructions of the thoracic cavity to assess tumour volumes pre‐ and post‐treatment with genetic circuits (PBS and CMV‐scrR, *n* = 5; CMV‐siR^E^, *n* = 6). (D) Representative H&E‐stained tumour sections. Scale bar: 100 μm. (E) Representative images of immunohistochemistry (IHC) staining for EGFR and Caspase‐3 proteins in lung sections. Scale bar: 100 μm. (F, G) Quantitative analysis of EGFR and Caspase‐3 proteins in immunohistochemically stained lung sections (*n* = 4 per group). (H) Quantitative real‐time polymerase chain reaction (RT–PCR) analysis of EGFR mRNA levels in tumour samples (*n* = 5 per group). (I, J) Western blot analysis of EGFR protein levels in tumour samples. Representative Western blots (I) and densitometric analysis data (J) are shown (*n* = 5 per group). The values are presented as the means ± SEMs. Significance was determined using one‐way analysis of variance (ANOVA) followed by Dunnett's multiple comparison test in panels (F–H) and (J). *p < .05; **p < .01; ***p < .005; ****p < .0001.

Another major concern about the siRNA treatment is the off‐target risk. We analyzed transcriptome changes in tumours and normal tissues, distinguishing between miRNA‐like off‐target effects and on‐target effects of EGFR silencing. We identified 165, 206, 307 and 111 significantly altered transcripts (mean reads > 500, fold change > 2 and *p* < .05) in the lung, liver, spleen and kidney respectively, in CMV‐siR^E^ circuit‐treated mice in comparison with the control ones (Figure 4A). Next, we evaluated whether the downregulated transcripts were directly targeted by EGFR siRNA in a miRNA‐like manner. No statistically significant enrichment for perfect matches between the EGFR siRNA seed region and 3′‐UTRs of downregulated transcripts was observed in normal tissues (Figure 4B). In contrast, tumour tissues from CMV‐siR^E^ circuit‐treated mice showed substantial transcriptome alterations (941 differentially expressed transcripts), with only two of the 269 downregulated transcripts having direct binding potential to the EGFR siRNA seed sequence. Gene ontology (GO) enrichment analysis in tumour cells revealed that six of the top 20 GO terms were closely linked to EGFR signalling (Figure 4C). This enrichment of GO terms associated with EGFR signalling pathways suggests that widespread transcriptome alterations primarily resulted from direct EGFR target silencing, accompanied by indirect blockade of EGFR downstream signalling cascades by the CMV‐siR^E^ circuit. The data indicates that the off‐target effects in normal tissues are minimal compared to the on‐target effects observed in tumour cells, supporting the specificity and safety of the CMV‐siR^E^ circuit in EGFR siRNA delivery. On the other hand, self‐assembled siRNAs utilize mammalian cellular machinery for gene regulation. The liver, central to EGFR siRNA‐encapsulating sEV production, raised concerns about the potential disruption to host RNAi machinery. Thus, concerns about hepatotoxicity, specifically perturbing miR‐122,^8^ a major hepatocyte‐specific miRNA, were addressed. Analysis of miR‐122 target genes post‐CMV‐siR^E^ circuit treatment revealed only 13 significant changes among 1566 potential targets (Figure 4D). Most miR‐122 targets exhibited similar expression patterns with CMV‐scrR circuit treatment (Figure 4D), indicating that in vivo self‐assembled EGFR siRNAs maintain physiological levels in the liver. This ensures proper processing and normal secretion and avoids competition with endogenous miRNAs for RISC loading.

**FIGURE 4:**
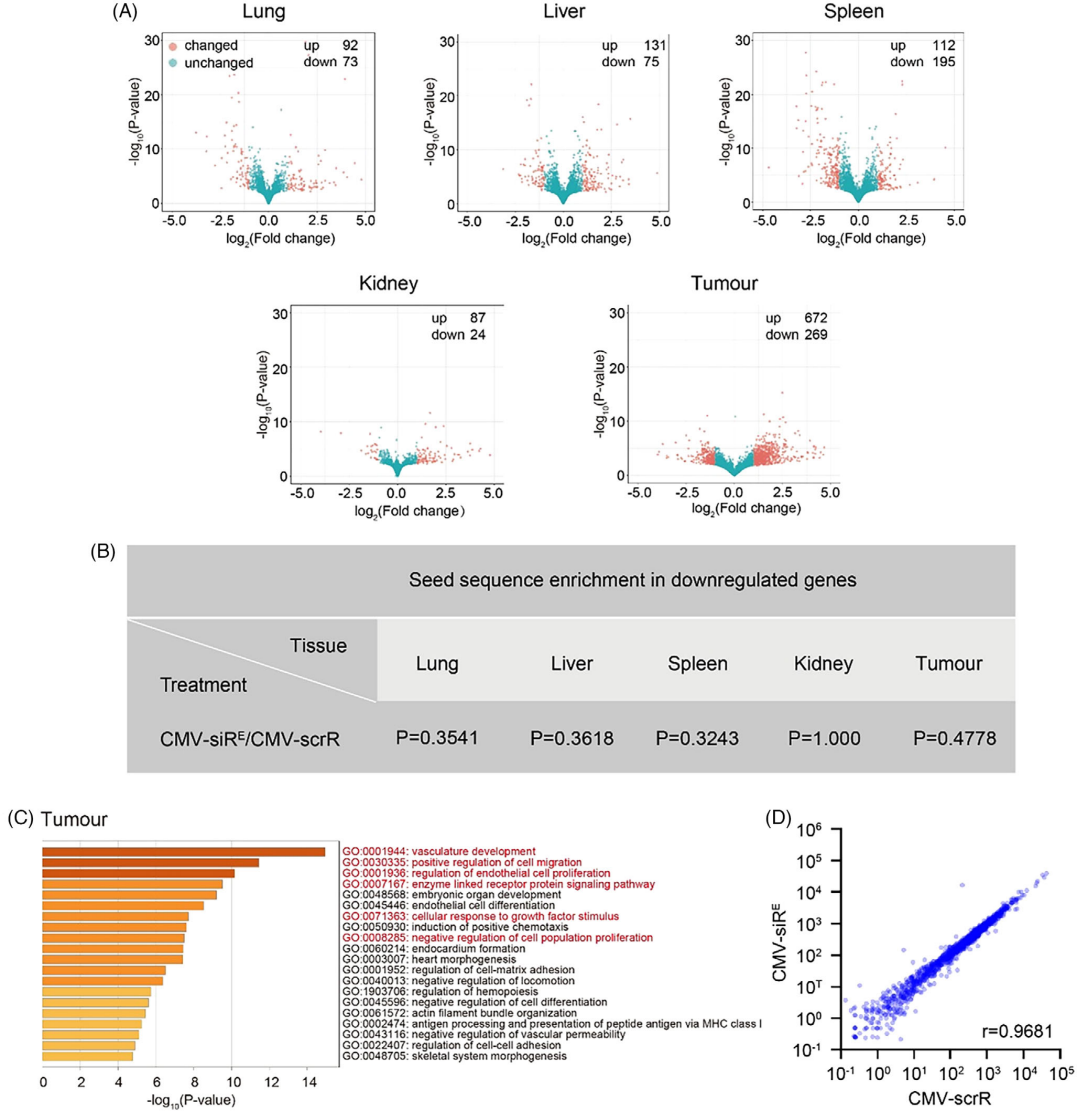
Assessment of miRNA‐like off‐target effects in an orthotopic lung cancer model after treatment with genetic circuits. Nude mice were intratracheally implanted with H1975 cells and analysed by micro‐CT at 25 days post‐inoculation to ensure the formation of tumours in the lungs. Mice were then intravenously injected with the cytomegalovirus (CMV)‐scrR or CMV‐siR^E^ circuit (10 mg/kg) every 2 days for a total of 7 injections. After treatment, mice were sacrificed, and normal tissues and tumours were harvested to determine the global transcriptome alterations by RNA sequencing. (A) Volcano plots showing global gene expression changes in normal tissues and tumours. The significantly altered genes (*p* < .05 and Log_2_FoldChange < −1 or > 1) in the CMV‐siR^E^ circuit‐treated groups compared with the CMV‐scrR‐treated group are marked in red. (B) Enrichment analysis of seed region (nucleotides 2−8 in the epidermal growth factor receptor (EGFR) small interfering RNA (siRNA) guide strand) complementarity with downregulated genes using Fisher's exact test. (C) Gene ontology (GO) analysis of the downregulated genes (*p* < .05 and Log_2_FoldChange < −1) in the CMV‐siR^E^ circuit‐treated groups (compared with the CMV‐scrR‐treated group). (D) Scatter plot showing the alterations of the target genes of miR‐122 in the liver after treatment with genetic circuits. *r*, Pearson correlation coefficient.

Our previous studies have proved the potential of a self‐assembled siRNA delivery system in disease treatment.^1^, ^2^, ^3^, ^4^ In this study, we comprehensively evaluated the potential on‐target and off‐target toxicity of self‐assembled EGFR siRNAs. In lung cancer models, we characterized the robust bioactivity and minimal side effects of self‐assembled siRNA delivery systems both in vitro and in vivo. Utilizing the self‐assembled siRNA delivery system, we provide a promising solution to the challenges of incorporating RNAi therapeutics into the medical realm: how to strike a delicate balance between efficient encapsulation of RNAi drugs and mitigating off‐target risks. Our research also provided a paradigmatic scheme for the evaluation of potential on‐ and off‐target toxicity which could benefit the design of in vivo self‐assembled siRNAs targeting any other genes.

## AUTHOR CONTRIBUTIONS

Xi Chen, Zhen Zhou, Xu Guo and Chen‐Yu Zhang conceived and designed the experiments. Hongyuan Guo, Yuanyuan Su, Xiao Hu, Hao Zhu and Ruoyan Zhang performed the experiments. Hongyuan Guo, Yuanyuan Su, Xu Guo and Zhen Zhou contributed the materials. Xin Yan analyzed all the results of H & E in a single‐blind manner. Hongyuan Guo and Zhen Zhou analysed the data. Xi Chen and Hongyuan Guo wrote the manuscript. All authors read and approved the final manuscript.

## CONFLICT OF INTEREST STATEMENT

The authors declare no conflict of interest.

## Supporting information

Supporting Information
