# Supplementary material for: Evaluation of on‐ and off‐target effects of self‐assembled epidermal growth factor receptor small interfering RNA delivery system
Source: Clin Transl Med. 2024 Feb 5;14(2):e1579. doi: 10.1002/ctm2.1579 (PMC10844837; doi:10.1002/ctm2.1579)
Supplement: Supplementary file 1 — Supporting Information [file CTM2-14-e1579-s001.docx]

**Supplemental Materials**

**This PDF file includes:**

**Materials and Methods**

**Supplementary Figure 1-6**

**Materials and Methods**

**Design and construction of the genetic circuits**

The coding sequence of human EGFR mRNA (NM_005228.4) was used to generate candidate siRNA with the BLOCK-iT™ RNAi Designer web tool by Thermo Fisher (https://rnaidesigner.thermofisher.com/rnaiexpress/). Twenty EGFR siRNA sequences were designed, and these siRNA sequences were intended to share no more than 15 contiguous base pairs with any gene other than EGFR. To mitigate off-target effects, the mouse transcriptome was obtained from the ENCODE database, and RNAhybrid was used to scan potential miRNA-like off-targets of the siRNAs in mouse mRNA 3’-UTR sequences with the following criteria: perfect binding of the siRNA seed regions (nucleotides 2–8 in the siRNA guide strand) to complementary sites in mRNA 3’-UTRs; a minimal folding energy of the hybrid of less than −25 kcal/mol; and a length of the internal loop and bulge loop not exceeding 5 bases.

The CMV-siR^E^ circuit was generated by inserting an EGFR siRNA sequence (5’-ATTTCTATCAATGCAAGCCAC-3’) into a 145-bp pre-miR-155 backbone (5’-CTGGAGGCTTGCTGAAGGCTGTATGCTGAATTCGATTTCTATCAATGCAAGCCACGTTTTGGCCACTGACTGACGTGGCTTGTTGATAGAAATCACCGGTCAGGACACAAGGCCTGTTACTAGCACTCACATGGAACAAATGGCC-3’). A circuit designed to express a scrambled RNA was used as the negative control.

The genetic circuits were constructed in the form of DNA plasmids and were dissolved in PBS without additional formulations. For *in vitro* cell transfection, the genetic circuits (plasmids) were transfected into cultured cells using Lipofectamine 2000 transfection reagent (Invitrogen, 11668019, MA, USA) according to the manufacturer’s instructions. For *in vivo* experiments, the genetic circuits were directly injected into mice via a standard tail vein injection protocol (injection of 200 μL of solution within 3 sec).

The constructed plasmids were transformed into competent *E. coli* DH5α cells (Tsingke, TSC01, Beijing), cultured in LB medium (containing 50 μg/mL spectinomycin) for 14 h at 37 °C in an incubator and isolated with an EndoFree Maxi Extraction and Purification Plasmid Kit V2 (Tiangen, DP120, Beijing, China) according to the manufacturer’s instructions. The purified plasmids were sequenced to ensure that the genetic circuits were constructed correctly.

**Cell culture**

Human lung adenocarcinoma cell line H1975, human bronchial epithelial cell line BEAS-2B and human embryonic kidney cell line HEK293T were purchased from the Shanghai Institute of Cell Biology, Chinese Academy of Sciences (Shanghai, China). HEK293T cells were cultured in DMEM (Gibco, C11995500BT, MA, USA) supplemented with 10% foetal bovine serum (FBS; Gibco, 10099141C, Australia), penicillin and streptomycin (Gibco, 15140122, MA, USA) in a 5% CO_2_ water-saturated atmosphere H1975 and BEAS-2B cells were cultured in RPMI 1640 medium (Gibco, C11875500BT, MA, USA) supplemented with 10% FBS, penicillin and streptomycin in a 5% CO_2_, water-saturated atmosphere.

**sEV isolation**

sEV isolation from cell culture medium. Cell-conditioned medium was collected from HEK293T cells cultured for 48 h with Opit-MEM. The collected medium was first subjected to a centrifugation step of 400 × g for 10 min at room temperature to pellet and remove cells. All following centrifugation steps were performed at 4 °C. Next, the supernatant was spun at 2,000 × g for 30 min to remove debris and apoptotic bodies. Then, to pellet and collect EVs, the supernatant was centrifuged at 15,000 × g for 30 min. The resulting EVs pellet was resuspended in a large volume of PBS buffer. To remove any remaining large EVs, the supernatant was passed through a 0.22 μm pore PES filter (Millipore). This supernatant (pre-cleared medium) was next subjected to ultracentrifugation at 120,000 × g for 4 h in a SW 45 Ti Rotor Swinging Bucket rotor (Beckman Coulter, Fullerton, CA) to sediment small EVs (sEVs). The crude sEV pellet was again resuspended in a large volume of PBS followed by ultracentrifugation at 120,000 × g for 4 h to wash the sample. The washed pellet was resuspended in ice-cold PBS. sEVs were further purified by high-resolution iodixanol density gradients fractionation according to the manufacturer’s instructions.

To obtain highly pure sEVs, iodixanol density gradient fractionation was performed. Iodixanol density media (36%, 30%, 24%, 18% and 12%) (Sigma-Aldrich, D1556, MO, USA) were prepared in cold PBS. Crude sEVs were mixed with the 36% iodixanol density media and added to the bottom of a centrifugation tube. The solutions of descending concentrations of iodixanol density media were carefully layered on top yielding the complete gradient. The centrifugation tubes were subjected to ultracentrifugation at 120,000 × g for 12 h at 4 °C. Next, 3 mL solution from the top, 5 mL solution from the middle- and 3.5-mL solution from the bottom of the gradient were sequentially transferred to new ultracentrifugation tubes. The solution transferred from each layer were diluted 6-fold with PBS and subjected to ultracentrifugation at 120,000 × g for 4 h at 4 °C. sEVs were suspended in PBS for the cellular incubation assay, transmission electron microscopy (TEM) or Dynamic light scattering (DLS).

**sEV incubation assay**

HEK293T cells were seeded into 10 cm Petri dishes, and 10 μg of the genetic circuits were transfected with Lipofectamine 2000 reagent (Invitrogen). After 36 h, the cell culture medium was harvested, and sEVs were isolated as described above. After incubation with sEVs for 36 h, total RNA or protein was isolated for quantitative RT–PCR or Western blot analysis.

**Electron microscopy**

sEVs were suspended in phosphate-buffered saline (PBS) and fixed with 2% paraformaldehyde (PFA). The sEV solution was applied to a copper grid, post-negatively stained with 2% phosphotungstic acid for 10 min, and then dried for 2 min under incandescent light. Excess liquid was removed from the grid using filter paper. The copper grid was stored at room temperature until it was observed and photographed under a transmission electron microscope (H-7650 Hitachi microscope; Hitachi, Tokyo, Japan).

**Dynamic light scattering**

The size and homogeneity distributions of the extracted sEVs were assessed through dynamic light scattering (DLS). The samples were diluted in sterile, particle-free PBS at a ratio of 1:200, mixed, and ultrasonicated to achieve uniform distribution of the particles before being introduced into the laser chamber. DLS measurements were conducted at 25 °C using the NanoBrook Omni (Brookhaven Instruments, USA).

**Immunoprecipitation**

Immunoprecipitation of serum extracellular RNAs with anti-CD9 antibody (Santa Cruz Biotechnology) or anti-CD63 antibody (Santa Cruz Biotechnology) was conducted to investigate whether circulating EGFR siRNA is enclosed by serum small extracellular vesicles (sEVs). Specifically, serum sEVs derived from mice injected with CMV-siRE circuit for three cycles were diluted with 100 μL of 1 × PBS (pH 7.4) and combined with 4 μg of anti-CD9 antibody, anti-CD63 antibody, or mouse IgG (Santa Cruz Biotechnology) antibodies in a detergent-free solution. Subsequently, the binding solutions were incubated overnight at 4 °C. To capture the RNA-CD9 or RNA-CD63 complexes, 100 μL of Pierce Protein A/G Agarose beads (Thermo Fisher Scientific) were washed with 1 × PBS, suspended in 100 μL of 1 × PBS, and then added to the samples, followed by a 2-hour incubation on a shaker at room temperature. The A/G Agarose resin was separated from supernatant by centrifugation at 2500 × rpm for 5 min at 4 °C and then washed three times with 1 × PBS. The immunoprecipitates and supernatants were each divided in half: one half of each sample was eluted in 5 × SDS sample buffer followed by SDS/PAGE, while the other half was eluted in 900 μL of Trizol and processed for RNA isolation.

**Quantitative RT–PCR analysis**

Total RNA was extracted from cultured cells or mouse tissues using TRIzol Reagent (Invitrogen, 15596018, MA, USA) according to the manufacturer’s instructions. Mature siRNAs were quantified by TaqMan miRNA assays using customized probes (Applied Biosystems, CA, USA) according to the manufacturer’s instructions. In brief, 0.5 μg of total RNA was reverse transcribed into cDNA using a TaqMan MicroRNA Reverse Transcription Kit (Applied Biosystems, 4366597, CA, USA) and a customized stem–loop RT primer (Applied Biosystems, CA, USA). The following thermal cycling conditions were used for reverse transcription: 16 °C for 30 min, 42 °C for 30 min, and 85 °C for 5 min. Real-time PCR was performed using TaqMan™ Universal Master Mix II (Applied Biosystems, 4440040, CA, USA) and a LightCycler 480 system (Roche, IN, USA). Reactions were performed in a 96-well optical plate under the following thermal cycling conditions: 95 °C for 5 min, followed by 40 cycles of 95 °C for 15 sec and 60 °C for 1 min. All reactions were run in duplicate. After the reactions were completed, the cycle threshold (C_T_) values were determined with LightCycler 480 software, and the mean C_T_ value was determined from the PCR data.

To quantify the absolute amounts of siRNA, synthetic single-stranded EGFR siRNA was serially diluted, and a standard curve was generated by quantitative RT–PCR. The content of EGFR siRNA in serum was calculated with reference to the standard curve and shown as the absolute concentration (fM).

mRNA was reverse transcribed with AMV Reverse Transcriptase (TaKaRa, 2621, Dalian, China) and reverse primers after purification of total RNA with TRIzol (Invitrogen, 15596018) according to the manufacturer’s instructions. In brief, 1 μg of total RNA was reverse transcribed into cDNA using a reverse primer and AMV reverse transcriptase under the following conditions: 16 °C for 15 min, 42 °C for 60 min, and 85 °C for 5 min. Next, real-time PCR was performed with the reverse transcription (RT) product, EvaGreen Dye (Biotium, 31000, CA, USA) and specific primers for EGFR (forward: 5’-AGGCACGAGTAACAAGCTCAC-3’; reverse: 5’-ATGAGGACATAACCAGCCACC-3’) and GAPDH (forward: 5’-GGAGCGAGATCCCTCCAAAAT-3’; reverse: 5’-GGCTGTTGTCATACTTCTCATGG-3’). Reactions were conducted under the following thermal cycling conditions: 95 °C for 5 min, followed by 40 cycles at 95 °C for 30 sec, 60 °C for 30 sec and 72 °C for 1 min. After the reactions were completed, the C_T_ values were determined, and the relative levels of mRNAs were normalized to that of GAPDH and calculated by the 2^−ΔΔCT^ method.

**Western blot analysis**

Cell samples were rinsed with PBS and lysed with RIPA lysis buffer on ice for 30 min. Tissue samples were homogenized in four volumes of RIPA lysis buffer in an automatic tissue grinder at 4 °C and lysed with RIPA lysis buffer on ice for 30 min. If necessary, samples were sonicated in an ice bath until lysis was complete. Cell lysates or tissue homogenates were centrifuged at 4 °C for 10 min (12000 × g). Supernatants were collected, and protein concentrations were determined with a Pierce BCA protein concentration assay kit (Thermo Scientific, 23225, CA, USA). Protein samples were separated by 10% SDS–PAGE and transferred onto PVDF membranes. The membranes were blocked for 1 h at room temperature with 5% skim milk (for analysis of phosphorylated proteins, the membranes were blocked for 1 h with 5% BSA) and incubated overnight at 4 °C with primary antibodies. The membranes were washed 4 times with 1 × TBST for 15 min each, incubated with the secondary antibody at room temperature for 1 h and rinsed with 1 × TBST 3 times for 10 min each. Antibody–protein complexes were detected with SuperSignal™ West Pico PLUS Chemiluminescent Substrate (Thermo, 34580, CA, USA) and visualized using a Tanon 5200 Multi detection system (Tanon, Shanghai, China). Protein bands were analysed with ImageJ. The following primary antibodies were used: anti-EGFR (1:1000, Proteintech, 18986-1-AP), anti-GAPDH (1:2000, Proteintech, 0494-1-AP), anti-rabbit IgG (1:2000, Proteintech, SA00001-2).

**Apoptosis assay**

Cells were washed twice in cold PBS and resuspended in Annexin V binding buffer at a concentration of 1 × 10^6^ cells/mL. Then, 100 μL of the cell suspension (1 × 10^5^ cells) was incubated with 5 μL of FITC-Annexin V and 5 μL of propidium iodide using an Apoptosis Detection Kit (BD Biosciences, CA, USA). Then, 400 μL of binding buffer was added. The flow cytometry was used to determine the apoptosis rate.

**Targeting EGFR in lung cancer models**

*Orthotopic lung cancer model harbouring EGFR with the T790M mutation (H1975).* Intratracheal injection was used to establish the orthotopic animal model of lung cancer. Nude mice were anaesthetized by injection of 1% pentobarbital sodium (5 mL/kg). An intravenous catheter (0.8 × 25 mm) was inserted into the trachea, and 40 μL of 2 × 10^6^ H1975 cells suspended in PBS mixed with 20 μL of Matrigel were delivered into the lung through the catheter. After 30 days, mice were monitored for lung tumour formation by noninvasive micro-CT scanning and randomly divided into five groups. The mice in four of the groups were treated with PBS, CMV-scrR or CMV-siR^E^ circuit (10 mg/kg) via tail vein injection every 2 days for 2 weeks (7 total injections). Mice were monitored for survival time and tumour growth after treatment. For tumour growth analysis, mice were subjected to micro-CT scanning at the end of the treatment period and sacrificed for collection of lung tissues. The obtained samples were analysed by H&E staining, IHC staining or molecular assay. Lung, liver, brain, heart and muscle specimens were fixed with 4% PFA for H&E staining and IHC staining.

*Micro-CT scanning.* Micro-CT scanning was performed to assess the growth of lung tumours, and 3-D pulmonary images were reconstructed based on the scanning data to differentiate tumours from blood vessels. In brief, micro-CT scanning was performed using a Hiscan XM Micro CT System (Suzhou Hiscan Information Technology Co., Ltd). The X-ray tube settings were 60 kV and 134 μA, and images were acquired at 50 µm resolution. A 0.5° rotation step through a 360° angular range with an exposure time of 50 ms per step was used. Mice were anaesthetized with a continuous flow of a 3% isoflurane/oxygen mixture and scanned in a supine position. The micro-CT data were batch-sorted, processed and reconstructed using Hiscan Reconstruct software (version 3.0, Suzhou Hiscan Information Technology Co., Ltd). Images of the reconstructed data were subsequently generated using Hiscan Analyzer software (version 3.0, Suzhou Hiscan Information Technology Co., Ltd), and tumour volumes were calculated using the CTan program (SkyScan) according to the manufacturer’s instructions.

**Histopathology and immunohistochemistry**

Tissue was fixed overnight in 4% PFA and embedded in paraffin. Sections generated with a tissue slicer were then transferred to slides, stained H&E and mounted. Then slides were scanned with Pannoramic MIDI, 3DHISTECH) and tumour loading was confirmed and interpreted by experienced pathologists.

IHC analysis was performed according to standard protocols. Before staining, sections were baked at 60 °C for 1 h, dewaxed with xylene and rehydrated through a graded ethanol series. High-pressure citric acid antigen retrieval solution was heated for antigen retrieval for approximately 5 min. Sections were incubated with an anti-EGFR, anti-Caspase-3, anti-PCNA, anti-p-AKT or anti-p-ERK antibody at room temperature for 60 min. The following primary antibodies were used: anti-EGFR (1:100, Servicebio, GB13084) and anti-Caspase-3 (1:1000, Servicebio, GB11009-1). Immunoreactions were detected with diaminobenzidine as the chromogenic agent for 3 min. Protein expression was assessed by experienced pathologists and quantified using Image-Pro Plus software.

**RNA sequencing and bioinformatic analysis**

Tissues were harvested from EGFR DEL19 mice treated with the CMV-scrR or CMV-siR^E^ circuit (10 mg/kg) 7 times over a 2-week period. RNA extracted with TRIzol reagent was used for global transcriptome analysis by Annoroad Co. (Beijing, China). Differential gene expression analysis was performed with the R package DESeq2. Significantly differentially expressed genes were identified as those with *P* < 0.05 and Log_2_FoldChange < -1 or > 1. The seed enrichment *P* value was calculated using Fisher’s exact test.

**Statistical analysis**

Details of the statistical tests used can be found in the figure legends. All statistical analyses were performed using commercial software (GraphPad Prism 8.3.0). Data were first tested for a normal distribution. Differences among groups were compared by one-way ANOVA or two-way ANOVA as indicated in the figure legends, and multiple comparison testing was conducted with Dunnett’s test. The *n* values indicate the numbers of samples used in the experiments. Data are shown as the means with error bars indicating the SEMs. Significance was assumed for **P* < 0.05, ***P* < 0.01, ****P* < 0.005, and *****P* < 0.0001 (NS, not significant).

**Supplementary Figures**


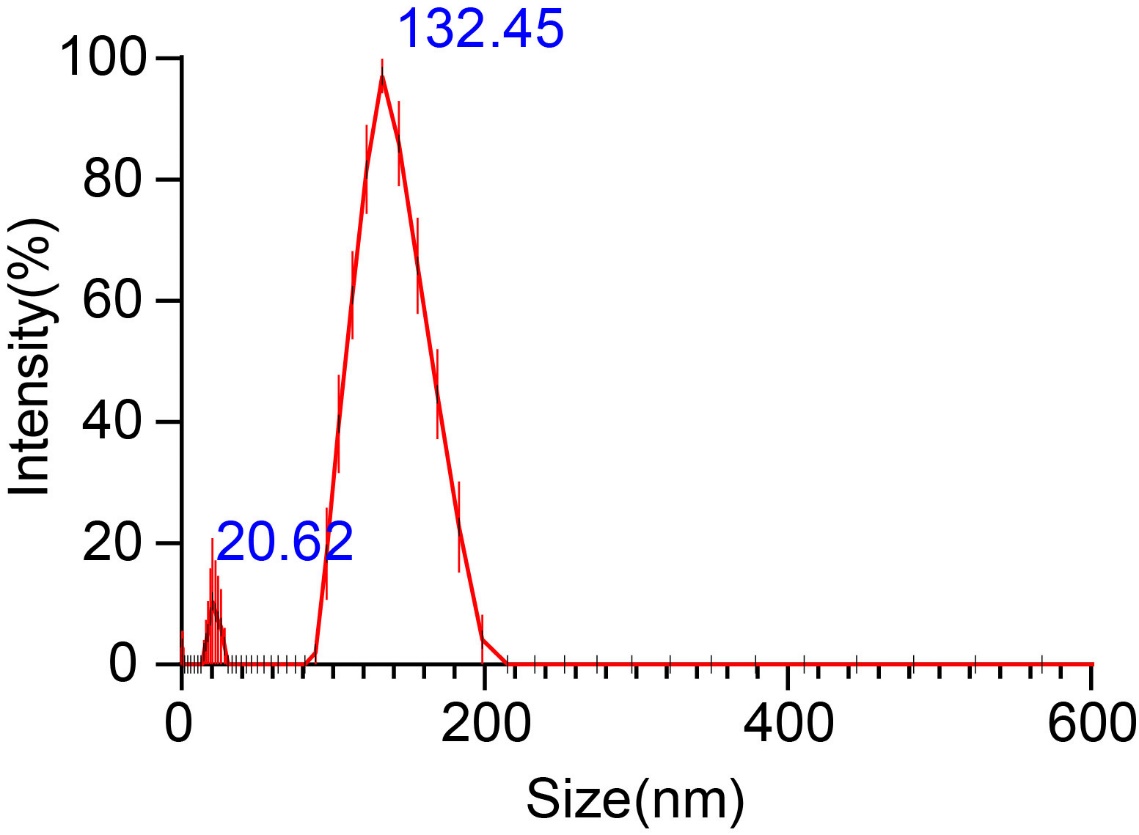


**Supplementary Figure 1. Size distribution histogram of sEVs obtained by dynamic light scattering (DLS).**


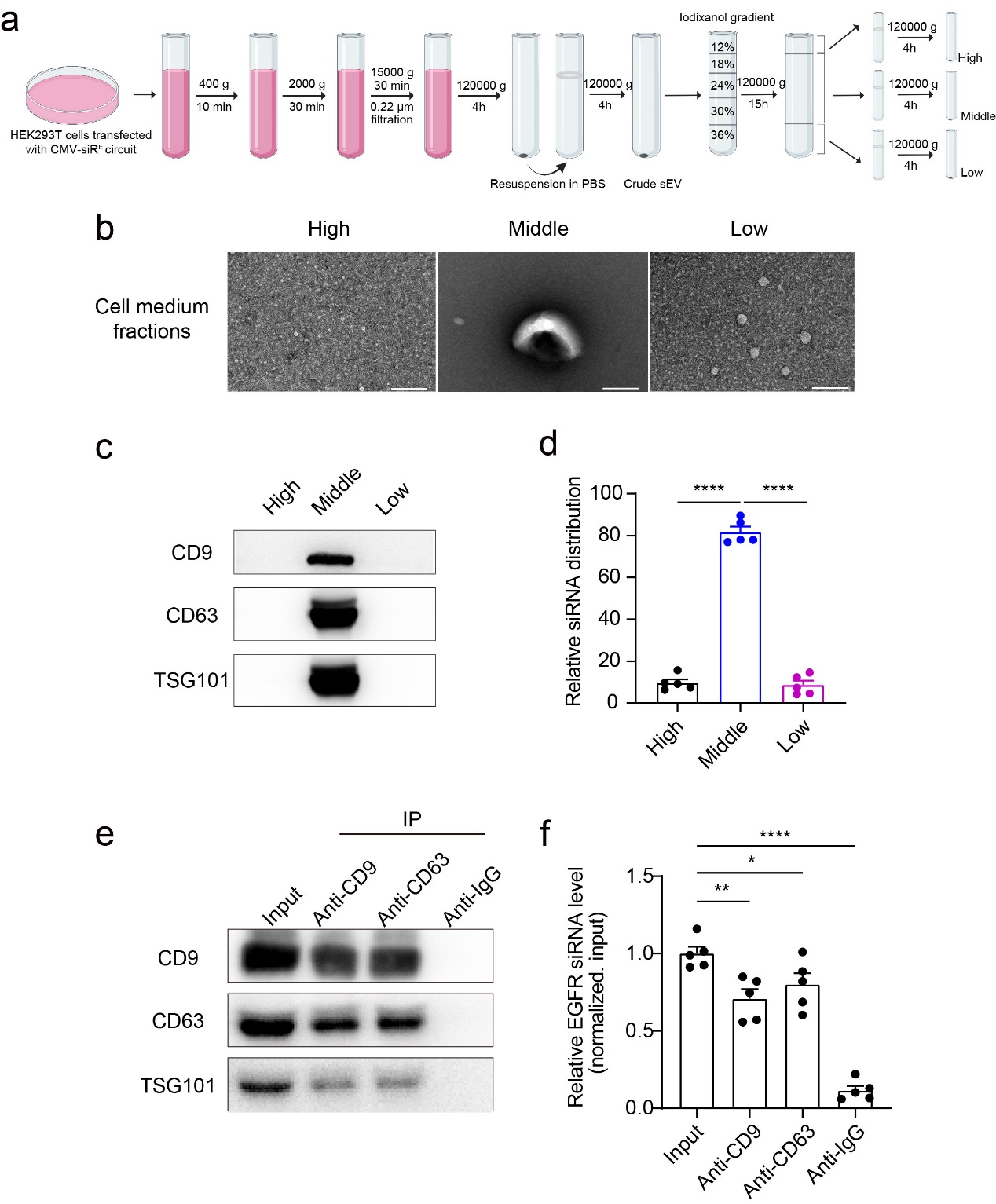


**Supplementary Figure 2. Characterization of the properties of siRNA-encapsulating sEVs.** HEK293T cells were transfected with CMV-siR^E^ circuit (2 μg per well in the 6-well plates). At 36 h post-transfection, crude sEVs were purified from the culture medium using differential centrifugation, and the membrane-enclosed sEVs were further separated from non-vesicular components using iodixanol density gradient centrifugation. **(a)** Schematic diagram of the separation of membrane-enclosed sEVs from non-vesicular components by high-resolution iodixanol density gradient centrifugation. **(b)** Negative stained TEM images of the low-, middle- and high-density fractions generated after iodixanol density gradient centrifugation. Scale bar: 100 nm. **(c)** Representative Western blots of the sEV membrane markers CD9, CD63 and TSG101 in different layers. **(d)** Quantitative RT–PCR analysis of the percentage of EGFR siRNA distribution in different layers. **(e)** Western blots probed with sEV protein markers (CD9, CD63 and TSG101) to assess the sEVs captured by Protein A/G Agarose beads following immunoprecipitation. Equal amount of sEVs was used as input. **(f)** Quantitative RT–PCR analysis of the percentage of EGFR siRNA distribution in immunoprecipitants. Values are presented as the means ± SEM. Signiﬁcance was determined using one-way ANOVA followed by Dunnett’s multiple comparison test in panel d and f. *****P* < 0.0001.


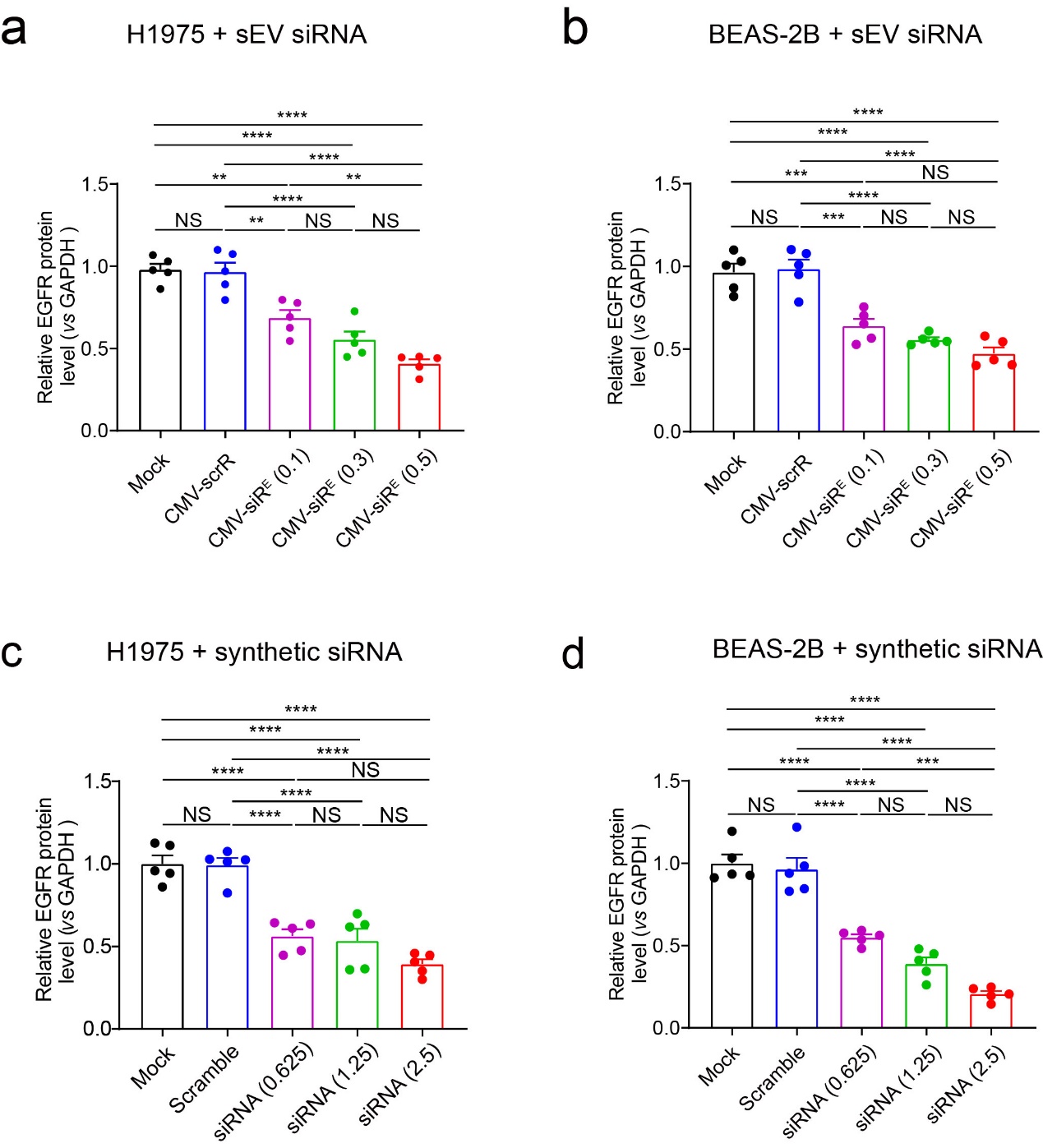


**Supplementary Figure 3. Quantitative analysis of EGFR protein level in cells incubated with self-assembled sEVs or transfected with synthetic EGFR siRNA. (a and b)** Densitometric analysis of western blot results of EGFR protein levels in H1975 and BEAS-2B cells incubated with sEVs derived from CMV-scrR or CMV-siR^E^ circuit-transfected HEK293T cells (n = 5 per group). Different doses (EGFR siRNA concentration in culture medium was 0.1, 0.3 and 0.5 fM, respectively) of CMV-siR^E^ derived sEVs were added to evaluate the dose-dependent effects. CMV-scrR derived sEVs (0.5 fM) were added as negative control, and untreated H1975 and BEAS-2B cells served as the mock controls. EGFR protein levels were determined 36 h post-incubation. **(c and d)** Densitometric analysis of western blot results of EGFR protein levels in H1975 and BEAS-2B cells transfected with synthetic EGFR siRNA (*n* = 5 per group). Different doses (EGFR siRNA concentration in culture medium was 0.625, 1.25 and 2.5 nM, respectively) of synthetic siRNA were added to evaluate the dose-dependent effects. EGFR protein levels were determined 36 h post-transfection. Synthetic scramble siRNA (2.5 nM) was transfected as negative control, and untransfected H1975 and BEAS-2B cells served as the mock controls. Signiﬁcance was determined using one-way ANOVA followed by Dunnett’s multiple comparison test in panels a-d. **P* < 0.05; ***P* < 0.01; ****P* < 0.005; *****P* < 0.0001; NS, not significant.


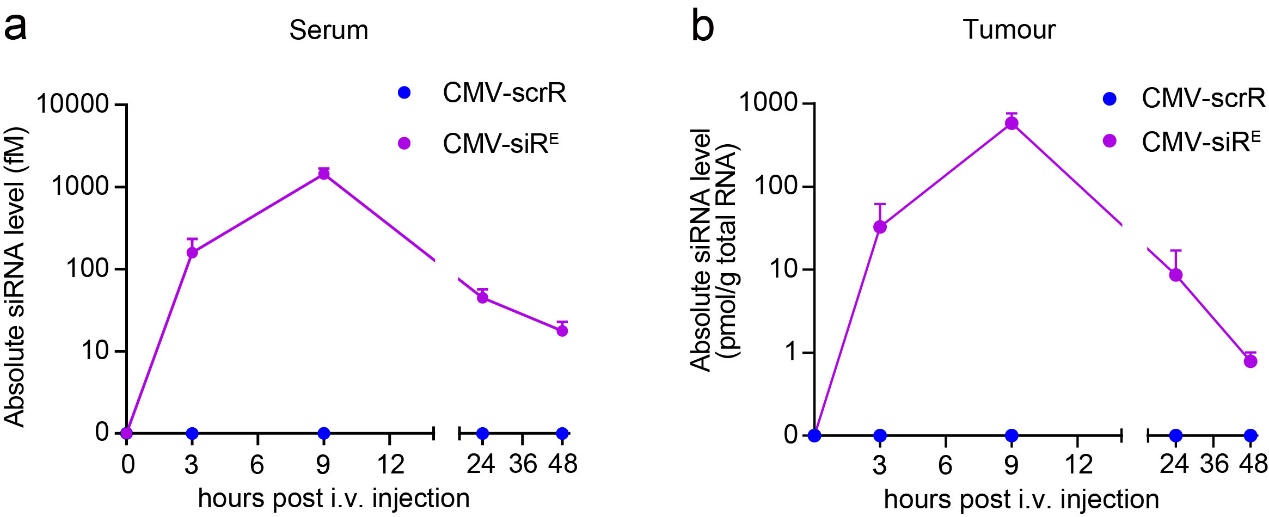


**Supplementary Figure 4. Kinetics of EGFR siRNA in the serum and tumour tissue after intravenous injection of the CMV-scrR or CMV-siR^E^ circuit (10 mg/kg) into mice (*n = 5* per group).**


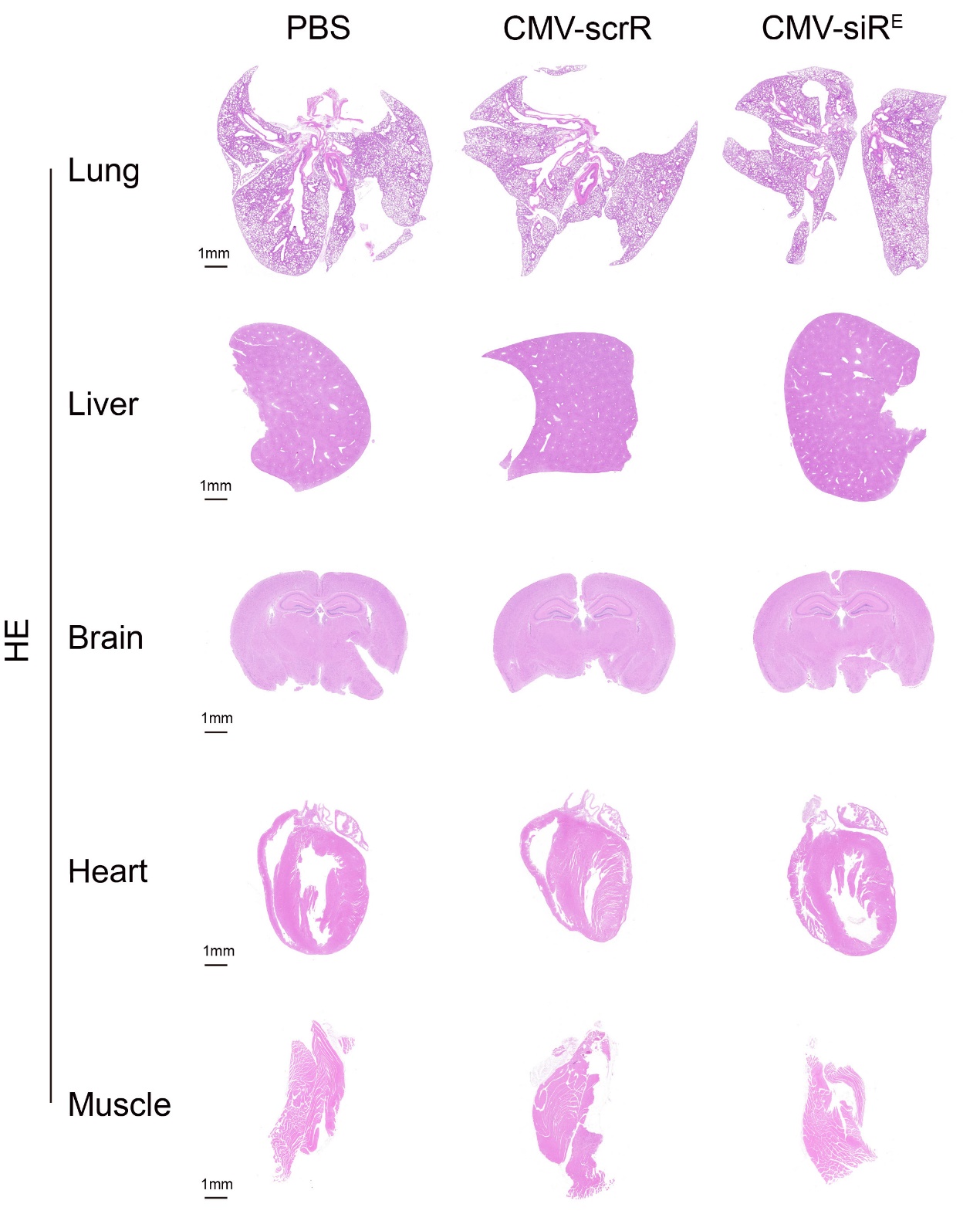


**Supplementary Figure 5. Evaluation of tissue damage in an orthotopic lung cancer model following intravenous injection of genetic circuits.** Nude mice were intratracheally implanted with H1975 cells and analysed by micro-CT at 25 days post-inoculation to ensure the formation of tumours in the lungs. Mice were then intravenously injected with PBS or the CMV-scrR or CMV-siR^E^ circuit (10 mg/kg) every 2 days for a total of 7 injections. After treatment, mice were sacrificed, and tissue samples were collected and analysed for tissue damage. Representative H&E staining of lung, liver, brain, heart and muscle tissues was shown. Scale bar: 100 μm.


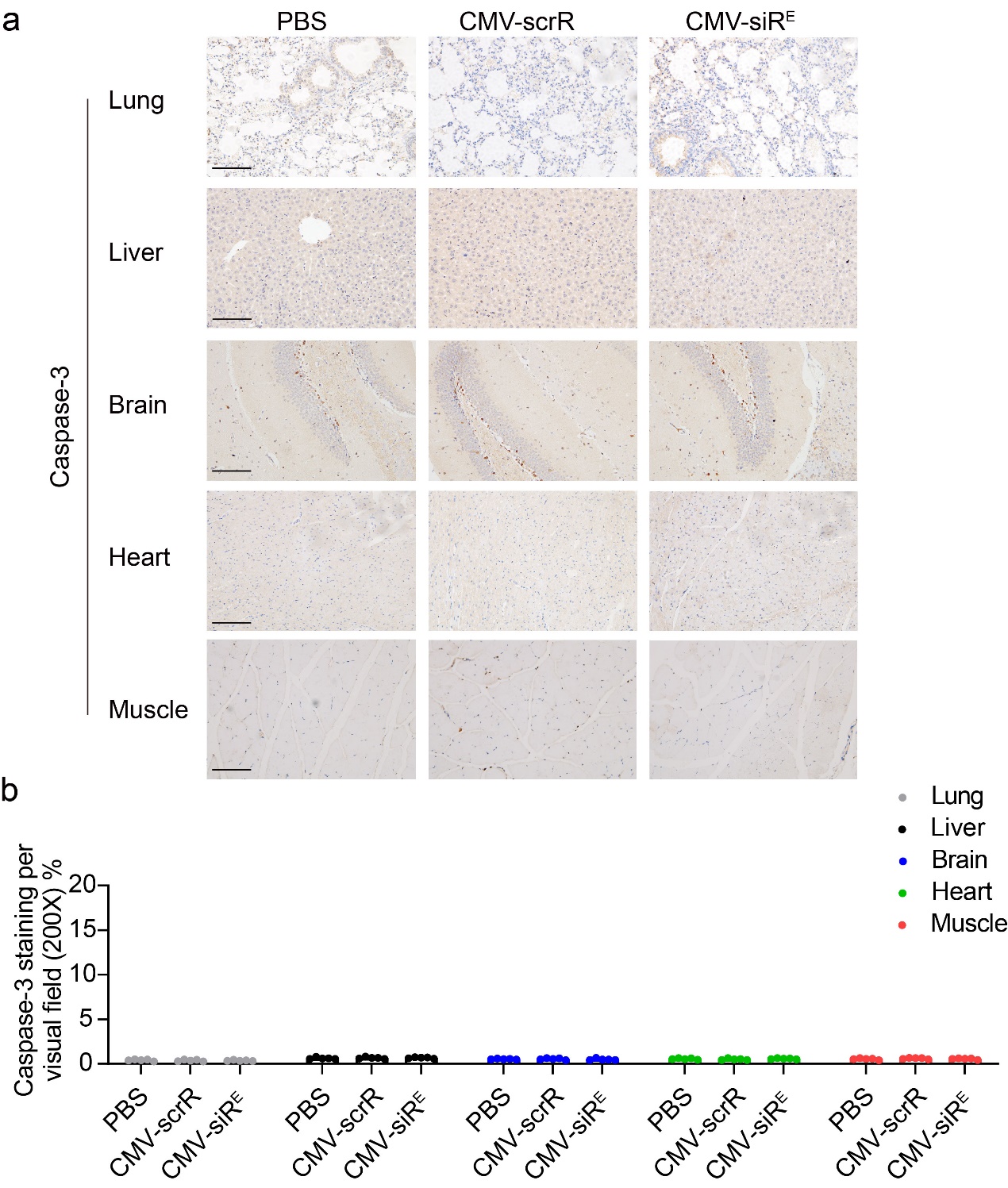


**Supplementary Figure 6.** **IHC staining for Caspase-3 proteins in tissue sections from an orthotopic lung cancer model following intravenous injection of genetic circuits.** Nude mice were intratracheally implanted with H1975 cells and analysed by micro-CT at 25 days post-inoculation to ensure the formation of tumours in the lungs. Mice were then intravenously injected with PBS or the CMV-scrR or CMV-siR^E^ circuit (10 mg/kg) every 2 days for a total of 7 injections. After treatment, mice were sacrificed, and the expression levels of Caspase-3 proteins were evaluated by IHC staining of tissue sections. **(a)** Representative image of IHC staining for Caspase-3 proteins in lung, liver, brain, heart and muscle sections. Scale bar: 100 μm. **(b)** Quantitative analysis of Caspase-3 proteins in immunohistochemically stained lung, liver, brain, heart and muscle sections (*n* = 4 per group). The values are presented as the means ± SEMs. Signiﬁcance was determined using one-way ANOVA followed by Dunnett’s multiple comparison test in panel b. NS, not significant; UD, undetectable.
